# Feasibility of a Peer-Led Asthma and Smoking Prevention Project in Australian Schools with High Indigenous Youth

**DOI:** 10.3389/fped.2017.00033

**Published:** 2017-03-02

**Authors:** Gabrielle B. McCallum, Anne B. Chang, Cate A. Wilson, Helen L. Petsky, Jan Saunders, Susan J. Pizzutto, Siew Choo Su, Smita Shah

**Affiliations:** ^1^Child Health Division, Menzies School of Health Research, Charles Darwin University, Darwin, NT, Australia; ^2^Children’s Health Queensland, Queensland University of Technology, Brisbane, QLD, Australia; ^3^Asthma Foundation NT, Darwin, NT, Australia; ^4^Primary Health Care Education and Research Unit, Western Sydney Local Health District, Sydney, NSW, Australia

**Keywords:** asthma, adolescent, Indigenous, airway inflammation, tobacco smoking, school-based service

## Abstract

**Background:**

The high global burden of asthma and tobacco smoking among Indigenous people may potentially be reduced by appropriate interventions that target prevention of tobacco smoke uptake and improved asthma management. The latter includes targeted treatment based on airway inflammation. We undertook a feasibility study in two Darwin schools with a high proportion of Indigenous youth to determine the feasibility of an innovative, peer-led, school-based education program called the Asthma and Smoking Prevention Project (ASPP). A subset of children with reported persistent respiratory symptoms were also clinically evaluated to determine the lower airway inflammatory profile and optimize asthma management.

**Methods:**

The ASPP is founded on an evidence-based three-step program and targets improving asthma management and preventing the uptake of tobacco smoking. The program uses a student-centered approach in which senior students (peer leaders) deliver the ASPP to Grade 7 students using activities, videos, and games. Students completed questionnaires related to asthma and smoking at baseline and 3 months after program delivery. Students with respiratory symptoms at 3 months were invited for a comprehensive clinical evaluation and tests including sputum induction.

**Results:**

The ASPP was well received. Of the 203 students involved, 56 (28%) were Indigenous and 70% completed baseline and follow-up questionnaires. Self-reported asthma was high (19%), 10% of students reported smoking and 63% reported exposure to tobacco at home. Of the 22 students who were clinically evaluated, 41% were Indigenous. Clinically important airway inflammation was high; 23% had Fractional Exhaled Nitric Oxide Levels ≥35 ppb, 88% had airway neutrophilia (>15%), and 29% had airway eosinophilia (>2.5%). Optimization of medication and management was required in 59% of students.

**Conclusion:**

Our study has demonstrated the implementation of the ASPP was well received by the schools as well as by the students. The high prevalence of clinically important airway inflammation and suboptimal asthma management highlights the need for a community-based study on persistent respiratory symptoms in adolescents to reduce the burden of chronic lung disease particularly for Indigenous Australians.

## Introduction

Respiratory health is poorer in Indigenous populations globally (compared to their non-Indigenous counterparts) including in Australia (1). Asthma and tobacco smoking are arguably the most important public health issues of all the respiratory health problems relevant to Indigenous populations (2, 3). Tobacco smoking is a major risk factor for adverse asthma-related outcomes (4). Further, despite declining rates of daily smoking in Australia (5), the prevalence of smoking among Indigenous people, with and without asthma remains disproportionately higher than other Australians (2). The prevalence of asthma is also higher in Indigenous Australians (17.5%) compared with non-Indigenous Australians (10.1%) (6), with poorer asthma outcomes more likely in Indigenous Australians (e.g., they are three times more likely to die from asthma) (7). However, both these respiratory health problems (tobacco smoking and asthma outcomes) can be improved by targeted effective programs that are culture specific (8, 9).

The various effective programs for tobacco control include legal, community, and individual approaches (10, 11). At the individual level, preventing the uptake of tobacco smoking is arguably superior to methods for quitting smoking as smoking behaviors are often established in youth and are a strong predictor of later daily smoking and poorer long-term outcomes (12, 13). In Australia, more than two-thirds of Indigenous smokers and ex-smokers began smoking regularly before the age of 18 years (6). Thus, to tackle the high prevalence of tobacco smoking in at-risk groups such as Indigenous people, prevention strategies in adolescence is important and needed.

However, there are few preventative programs targeting at-risk groups such as Indigenous populations (14). In non-Indigenous settings, current evidence suggests that school-based smoking prevention programs can have substantial positive effects in both the short and long term (11). Schools are an ideal place for initiating health promotion programs as they provide easy access to the target group and complement health and well-being. They also encourage young people to assume leadership roles and take responsibility for their health. Peer-led education also provides a unique opportunity to disseminate hard to deliver messages. At this age, peers have a greater influence on health behaviors than do parents or health personnel (15).

A peer-led program, the “Adolescent Asthma Action Program” (Triple A), was developed as a response to concerns about high rates of asthma attacks, school absenteeism and smoking in schools (15). Triple A was efficacious in non-Indigenous settings in Australia (16) and Jordan (17), but has not been available in schools with at-risk groups such as Indigenous youth. We, therefore, conducted a feasibility study based on the Triple A program (15, 16) with an added smoking prevention module, called the “Asthma and Smoking Prevention Project” (ASPP).

Other strategies to improving respiratory health (e.g., asthma outcomes) include targeted therapies based on airway inflammation (18). Non-eosinophilic airway inflammation, measured by sputum cellularity (19), is an important cause of severe asthma (20) and is associated with smoking (21). While eosinophilic asthma responds to corticosteroids, non-eosinophilic asthma has a poor response to corticosteroids and is related to increased asthma severity (22). An important association with non-eosinophilic airway inflammation includes systemic inflammatory markers (e.g., C-reactive protein and interleukin-6) (23). These drivers of inflammation are modifiable (23) and have the potential to improve detection and management of respiratory health. None of these factors, however, have been examined in adolescents with persistent respiratory symptoms.

Thus, in our feasibility study, our primary aim was to determine whether the ASPP was acceptable and relevant for schools in Northern Territory (NT) schools with high proportion of Indigenous students. Our secondary aim was to clinically evaluate a subset of students with self-reported respiratory symptoms to (i) examine the type of lower airway inflammation profile and (ii) optimize asthma management.

## Materials and Methods

### Study Design and Setting

We conducted this feasibility study (during March to June 2014) in two schools with a high number of Indigenous students in Darwin, the capital of the NT of Australia. A third school with more than 170 Grade 7 students withdrew prior to implementing ASPP due to logistical issues at the school level unrelated to the study. One school was a middle school (Grades 7–9) and the second a high school (Grade 7–12). This study was approved by the local Human Research Ethics Committee (HREC-2012-1900) and the NT Department of Education and Children’s Services (DET2013/138). One school used the opt-out format for consent, whereas the second requested the opt-in approach where written informed consent was obtained from caregivers. Indigenous ethnicity was self-reported (Aboriginal and/or Torres Strait Islander). Participants in our study were minors, thus consent was required to be obtained from caregivers.

### Implementation of the ASPP

High-level negotiation was undertaken with the NT Department of Education and Children’s Services to support the implementation of the ASPP into the nominated schools, and with school principals to embed the ASPP into the Health and Physical Education curriculum. The principals also identified key teachers to help drive the implementation into Grade 7 classes. ASPP staff presented the program to key teaching staff prior to roll out of the ASPP.

### Participants

The ASPP participants consisted of two groups (i) Peer Leaders (15–17 years) and (ii) Grade 7 (12–14 years) students (the target intervention group). Peer Leaders were students in either Grade 9 or 11 (depending which school they attended). The number of Peer Leaders was dependent on the size of the school. Selection of Peer Leaders was undertaken by teachers, based on assessment of student leadership qualities. All Grade 7 students completed the program, but the exhaled carbon monoxide (eCO) test and questionnaires were obtained only in those with a completed consent form.

### Details of the ASPP

We briefly describe the Triple A (15, 16) methods as they have previously been published (15, 17, 24). The ASPP is based on the Triple A program (15, 16) enriched with experience from existing tobacco education programs for Indigenous Australians, including normative education, training and social consequences of smoking, parental influences, and self-efficacy to resist smoking (25). The ASPP uses a student-centered strength-based approach to increase knowledge of asthma at a school level, create a supportive environment for students with asthma, and to promote a non-smoking culture in schools.

The theoretical basis of ASPP is embedded in social cognitive theory (15), which proposes a reciprocal interaction between a person, a targeted behavior, and a particular social context. It emphasizes that people learn not only from their own experiences but also by observing the actions of others. Adolescents are encouraged to (i) observe and imitate the positive behaviors of others, (ii) see positive behaviors modeled and practiced, (iii) empower and increase their own capability and confidence to implement new skills, (iv) adopt positive attitudes about implementing new skills, and (v) experience a supportive environment in order to use their new skills. The ASPP has been shown to encourage a sense of personal responsibility and improved quality of life for students with asthma, with fewer asthma attacks and reduced school absenteeism (17).

#### Educator Workshop and Steps of the ASPP

**Figure 1:**
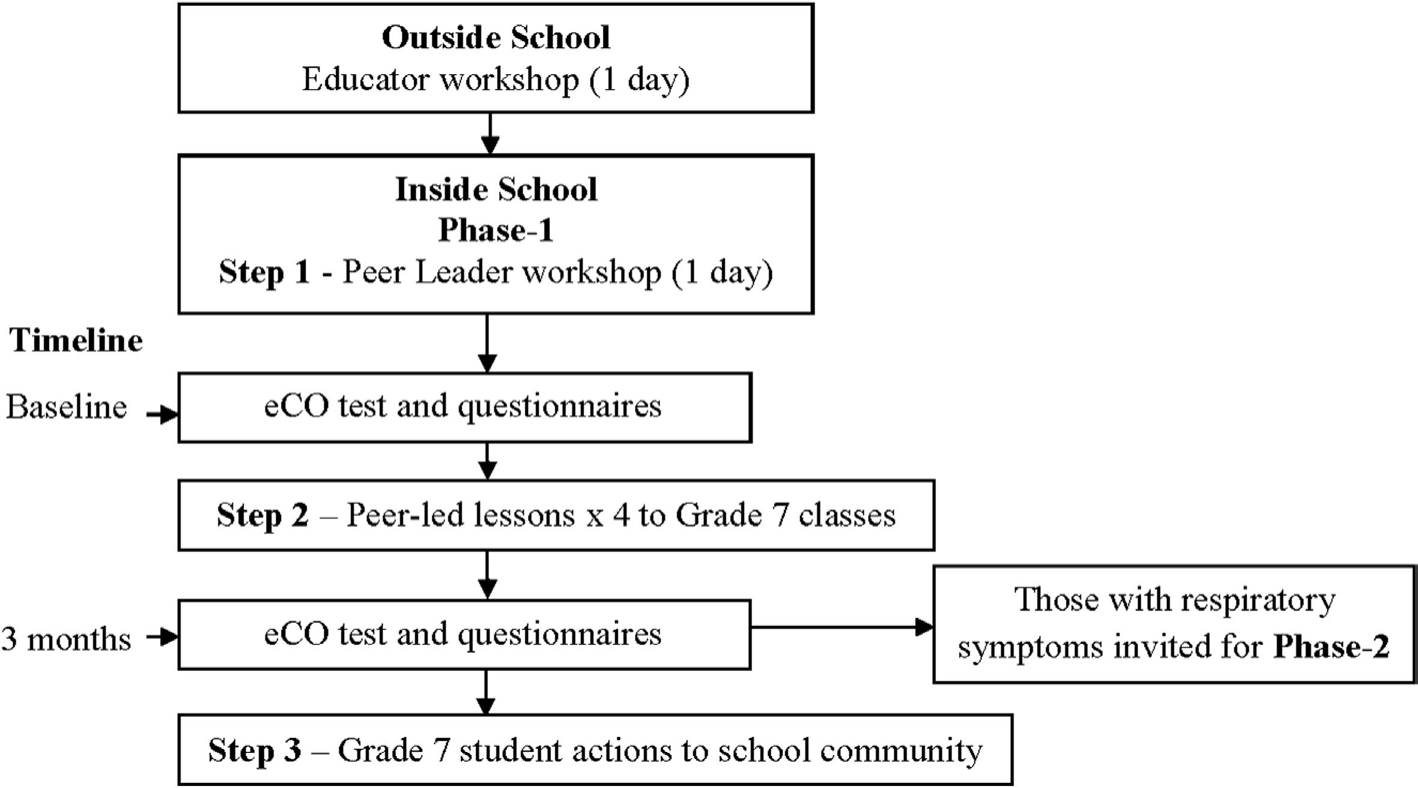
**Asthma and Smoking Prevention Project flow diagram**.

Before implementation of the ASPP in schools, educators were trained in an interactive 1-day workshop using standardized program manuals. The educators included Asthma Foundation NT staff, school nurses, university students (e.g., pharmacy students) (26), and research nurses. Following the educators’ workshop, the school component was undertaken and consisted of three steps (Figure 1) (16).

##### Step 1

Involved the ASPP educators’ training volunteer students were involved as peer leaders in an interactive 1-day workshop. The process was based on empowerment education, using a variety of strategies (e.g., videos, games, role plays, and quiz shows) during school lessons. Peer leaders learnt about asthma and smoking refusal skills and strategies to lead and teach their younger peers using standardized program manuals, detailed lesson plans, and materials (15).

##### Step 2

The peer leaders worked in groups of four (with support from class teachers) and delivered the four lessons of the ASPP to Grade 7 classes (target group) using standardized program manuals and materials (15). Sessions focused on asthma (how to avoid triggers, prevent exercise induced asthma, and what to do during an asthma attack) and peer pressure resistance training (e.g., how to say “no” to smoking; taking a smoke-free pledge). Learning was through videos, interactive games, and quiz shows, while furthering their skills in group leadership (15).

##### Step 3

The Grade 7 students disseminated what they had learnt in the ASPP to members of the school community and family through creative performances, e.g., drama, dance, and rap activities.

### Data Collection

At baseline (before the ASPP was delivered), students (who consented) undertook an eCO test and questionnaire. The eCO test was done using the PiCO^+^ Smokerlyzer^®^ device [Bedfont Scientific, England, UK measuring in parts per million (ppm)]. In our study, the cut off point for adolescent smokers was defined as elevated levels of carbon monoxide in breath (CO) >4 ppm (27). Student questionnaires included: the International Study of Asthma and Allergies in Childhood Questionnaire (28) with modified questions for chronic respiratory disease from the St. George’s Respiratory Questionnaire, questions on cough (29), the asthma control test (ACT) was a composite score (ranging from 0 to 12) and smoking-related questions. eCO and questionnaires were repeated at the 3-month follow-up.

At the end of their workshop, Peer Leaders completed a feedback questionnaire. Teaching staff also provided informal feedback about the ASPP.

### Clinical Evaluation

Students reporting current respiratory symptoms or self-reported asthma on the 3-month follow-up questionnaire were invited to have a clinical evaluation by a respiratory specialist. Demographic, medical history, and clinical data were recorded on standardized data collection forms for students whose caregiver provided written consent.

Participants were clinically evaluated and undertook additional tests: Fractional Exhaled Nitric Oxide (FE_NO_) using the Niox Mino (Aerocrine, Sweden) (30), spirometry (easy on-PC, ndd, Zurich, Switzerland), airway hyper-responsiveness (AHR) using hypertonic saline and sputum induction. The standardized 4.5% hypertonic saline challenge was used and sputum was processed using standard methods as previously done and (31) venipuncture (for full blood count and C-reactive protein) was also undertaken in those who had provided consent.

### Definitions

An AHR positive result was defined as a fall in forced expiratory volume during the first second (FEV_1_) of ≥15% from baseline (31). FeNO values ≥35 ppb (30) were considered abnormal. Airway eosinophilia and neutrophilia were considered present when the percentage of sputum eosinophils and neutrophilia were >3 and >15%, respectively (32, 33). Optimization of clinical management and classification of asthma was in accordance with the Australian Asthma Handbook. Management and classification for cough was based on an evidence-based cough guideline (34).

### Analyses

Data were entered on an Access database and analyzed using Stata version 14 (StataCorp, College Station, TX, USA). Data are presented as numbers and percentages, median and interquartile range (IQR) 25–75%, mean and SD or range, depending on data distribution. As this was a feasibility pilot study, before and after statistical comparison and a sample size calculation were not undertaken. However, we aimed to recruit 200 Grade 7 students as our target group. Qualitative data from Peer Leaders were thematically described.

## Results

### Schools/Demographics

Of the total number of Grade 7s (School 1: *n* = 190 and School 2: *n* = 77), consent was obtained from 49% from School 1 and 98% from School 2. A total of 203 students from the two schools participated in this study (e.g., completed questionnaires and eCO). The number of participating students per school is summarized in Table 1. Over half the students were female (55%) and 56 (28%) were Indigenous. Due to small numbers in this study, data were combined and results presented as a total.

**Table 1:** **School demographics of participating students**.

	School 1	School 2	Total
	
	*n* = 130 (%)	*n* = 73 (%)	*n* = 203 (%)
Male/female	61/69	30/43	91/112
Indigenous	19 (15)	37 (51)	56 (28)
Number of Grade 7s	93 (72)	66 (90)	159 (78)
Number of peer leaders	37 (28)	7 (10)	44 (22)
Completed baseline questionnaire^a^	108 (83)	68 (93)	176 (87)
Completed follow-up questionnaire^b^	107 (82)	49 (67)	156 (77)
Completed both questionnaires	98 (75)	44 (60)	142 (70)

*^a^n = 27 did not complete questionnaire*.

*^b^n = 47 did not complete questionnaire*.

### Questionnaires

Questionnaires were completed by 173 students at baseline and a further 156 at 3 months. Students completed baseline questionnaires unsupervised. As these were poorly completed (e.g., inconsistent and incomplete answers), ASPP staff supervised the completion of the follow-up questionnaires at 3 months (ASPP staff were in the classroom and helped when requested). At baseline, self-reported asthma was high (*n* = 33, 19%) with 20% of students reporting wheezing in the previous 12 months. Previous smoking was reported in 18 (10%) students, with first time smoking in 14 (8%) students aged less than 12 years. More than 24% reported that their friends were current smokers. Exposure to tobacco smoke at home was very high, with more than 99 (63%) students living in a house with >1 smoker (range 1–20 smokers). The smoking pledge was signed by 87 (49%) students, with 48 (27%) students leaving the question empty. Results of baseline and 3-month questionnaires are summarized in Table 2.

**Table 2:** **Baseline and follow-up questionnaire results**.^d^

	Baseline	Follow-up
	
	*n* = 176	*n* = 156
**ISAAC questionnaire**		
Wheezing in last 12 months	36 (20%)	34 (22%)
Wheezing during exercise in the last 12 months	41 (23%)	34 (22%)
Ever diagnosed with asthma by doctor	33 (19%)	21 (13%)

**Asthma control test (ACT)^a^**		
ACT score (0–12)^b^	9 (6–10)	9 (7–9)

**Cough**		
Any cough present	56 (32%)	30 (19%)
Acute cough (≤14 days)	32 (18%)	20 (13%)
Chronic cough (≥28 days)	11 (6%)	7 (4.5%)

**Smoking questions**		
Number of students ever tried smoking?	18 (10%)	15 (10%)
How old when tried smoking		
≤12 years	14 (8%)	13 (8%)
>12 years	7 (4%)	5 (3%)

**Family/household smoking**		
How many people smoke cigarettes at home? (range)	1–20	1–15

**Who smokes in your family?**		
Mum	48 (27%)	46 (29%)
Dad	63 (36%)	51 (33%)
Siblings (brother/sister)	31 (18%)	22 (14%)
Aunt/uncle(s)	33 (19%)	25 (16%)
Grandparent(s)	28 (16%)	24 (15%)
Other	24 (14%)	21 (13%)

**Are there rule about smoking cigarettes at home?^c^**		
No rules	27 (15%)	22 (14%)
No one is allowed to smoke in my home	62 (35%)	60 (38%)
Only special guests are allowed to smoke in my home	7 (4%)	2 (1%)
People are allowed to smoke in certain areas in my home	9 (5%)	16 (10%)
People are allowed to smoke anywhere in my home	7 (4%)	2 (1%)
People are allowed to smoke outside only	86 (49%)	75 (48%)

**Smoking and friends**		
**How many of your closest friends smoke?**		
None	102 (58%)	88 (56%)
1 or 2	24 (14%)	25 (16%)
3 or more	19 (11%)	14 (9%)
Unsure	19 (11%)	24 (15%)

**Would you smoke a cigarette, if friend offered it?**		
Yes	1 (1%)	2 (1%)
No	160 (91%)	143 (92%)
Maybe	8 (5%)	7 (4.5%)

*^a^Only students who self-reported asthma on questionnaires*.

*^b^Interquartile range (IQR) (25, 75)*.

*^c^Some responses have more than one answer*.

*^d^The numbers reported in the table do not match up (e.g., response to “ever tried smoking” was reported as no and response to “how old when smoking” an age was reported). We have, therefore, reported what was transcribed by the students and recognized as a limitation as described in the discussion*.

*Missing data at baseline: wheezing in last 12 months; *n* = 1 (0.5%), wheezing when exercising; *n* = 1 (1%), doctor confirmed asthma; *n* = 2 (1%), how bad is asthma today; *n* = 6 (3%), is asthma a problem when exercising; *n* = 6 (3%), cough because of asthma; *n* = 7 (4%), wake at night because of cough; *n* = 6 (3%), current cough; *n* = 6 (3%), ever tried smoking; *n* = 6 (3%), age tried smoking; *n* = 2 (1%), current smokers; *n* = 5 (3%), last cigarette; *n* = 5 (3%), how often smoke; *n* = 11 (6%), mum smoke; *n* = 9 (5%), dad smoke; *n* = 9 (5%), brother/sister smoke; *n* = 9 (5%), aunt/uncle; *n* = 9 (5%), grandparent; *n* = 9 (5%), other; *n* = 9 (5%), rules for smoking; *n* = 14 (8%), no smoking at home; *n* = 15 (9%), guests smoke’ *n* = 15 (9%), smoke in certain areas; *n* = 15 (9%), smoke anywhere; *n* = 15 (9%), smoke outside only; *n* = 15 (9%), friends smoke; *n* = 9 (5%), offered smoke; *n* = 7 (4%)*.

*Missing data at follow-up: wheezing in last 12 months; *n* = 1 (1%), wheezing when exercising; *n* = 1 (1%), doctor confirmed asthma; *n* = 1 (1%), how bad is asthma today; *n* = 1 (1%), is asthma a problem when exercising; *n* = 1 (1%), cough because of asthma; *n* = 1 (1%), wake at night because of cough; *n* = 1 (1%), current cough; *n* = 2 (2%), ever tried smoking; *n* = 3 (2%), age tried smoking; *n* = 1 (1%), current smokers; *n* = 3 (2%), last cigarette; *n* = 4 (3%), how often smoke; *n* = 8 (5%), mum smoke; *n* = 6 (4%), dad smoke; *n* = 6 (5%), brother/sister smoke; *n* = 6 (4%), aunt/uncle; *n* = 6 (4%), grandparent; *n* = 6 (4%), other; *n* = 7 (4.5%), rules for smoking; *n* = 10 (6%), no smoking at home; *n* = 10 (6%), guests smoke’ *n* = 10 (6%), smoke in certain areas; *n* = 10 (6%), smoke anywhere; *n* = 10 (6%), smoke outside only; *n* = 10 (6%), friends smoke; *n* = 5 (3%), offered smoke; *n* = 4 (3%)*.

Of those students who completed both questionnaires (*n* = 142, 70%), results were paired. Fifteen students completed the ACT; there was no change in the median ACT score from baseline (IQR 8.5, 6–10) to 3 months (IQR 8.5, 7–10). Only three students (2%) reported being current smokers at baseline with none at follow-up. Students’ exposure to tobacco smokers at home was high (range 1–20 smokers).

The eCO test was well received by students and was obtained for 91 (45%) students at baseline and 77 (34%) at 3 months. At baseline, elevated CO levels were found in three (3%) students compared with four (5%) at 3 months. Of the three students who reported being current smokers at baseline, only one had elevated CO levels consistent with that of a smoker (>4 ppm) (27).

### Feedback of the ASPP

The students’ feedback was overwhelmingly positive, with the most common responses described in Table 3. While we did not formally evaluate the ASPP for teaching staff, teachers were also receptive to the program as described: “*I am glad you let us trial this at our school. It has been a character building event for a lot of the Year 9’s that participated. We have noticed more engagement and involvement with other students and many have kept their connections to their Year 7 peers too. A great program, we hope you will consider us again*.” Further, both schools provided support for further implementation of the ASPP and have invited us to run the program again in the future.

**Table 3:** **Peer leader feedback**.

Questions	Most common responses
What are the most important points gained from Asthma and Smoking Prevention Project?	How important asthma awareness is (9%)
	How to treat someone who is having an asthma emergency (53%)
	How to teach others about asthma (9%)
	Asthma can kill you (2%)
	Asthma is important and serious (24%)
	Smoking makes asthma worse (4%)
	Ways to say no to smoking (9%)
	Go get check-ups often (2%)
	What causes asthma attacks (triggers) and how to deal with them (44%)
	What happens to your lungs when you have an asthma attack (4%)
	Asthma won’t prevent you from living your life (4%)
	Fun and teamwork (20%)
	Gaining confidence to speak in public (20%)
	How to say “no” to smoking (4%)
	Working together as a team (4%)

What did you like in particular?	The fun of the workshop (13%)
	Hands on activities/games (42%)
	How the presenters did the workshop—it was not boring/they had games (11%)
	Sit down and talk and then break the day into activities (2%)
	Role playing (2%)
	The videos about asthma (4%)
	Doing first aid presentations (6%)
	The safe environment for learning (4%)
	The straw game—it helped understand what it feels like to have asthma (4%)
	Learning about smoking (4%)

What can be improved?	Nothing (64%)
	More standing or more activities outside (7%)
	Structure of questionnaires could be improved (2%)

### Clinical Evaluation

Of the 37 students who reported current respiratory symptoms on the 3-month questionnaire, consent was obtained for 24 (65%). The clinical evaluation took place within 3 weeks of the 3-month questionnaire. Two students were away at the time leaving 22 students who undertook this component. Table 4 provides the demographic and clinical characteristics of these students.

**Table 4:** **Demographic and clinical characteristics of 22 students with current respiratory symptoms**.

	*n* = 22 (%)
Male/female	4/18
Age (years)^a^	13 (1.4)
Indigenous	9 (41%)
Gestational age (weeks)	40 (38–41)
Birth weight (kg)	3.2 (2.8, 3.6)
Special care nursery admission	1 (6%)
Previous respiratory hospitalization	3 (14%)
Mother smoked during pregnancy	7 (32%)
Exposed to household smoke	8 (36%)

**Symptoms**	
**Current symptoms**	
Breathless	6 (27%)
Wheeze	5 (23%)
Hemoptysis	0 (0%)
Chest pain	5 (23%)
Tiredness/lethargy	6 (27%)

**Ever symptoms**	
Breathless	15 (68%)
Wheeze	15 (68%)
Hemoptysis	1 (5%)
Chest pain	15 (68%)
Tiredness/lethargy	17 (77%)

**Clinical examination**	
Dry/wet cough	19/3
Chest wall hyperinflation	2 (9%)
Wheeze present	1 (5%)
Crackles present	0 (0%)
Chronic suppurative otitis media	1 (5%)

**Classification of persistent asthma**	
Mild	6 (27%)
Moderate	2 (9%)
Severe	1 (5%)

**Spirometry and FeNO (*n* = 22)**	
FEV_1_^b^	90 (82, 100)
Forced vital capacity^b^	95 (83, 102)
FE_NO_ <20 ppb	13 (59%)
FE_NO_ 20–<35 ppb	4 (18%)
FE_NO_ ≥35 ppb	5 (23%)

**Sputum characteristics (*n* = 17)**	
Mucoid	3 (18%)
Mucopurulent	1 (6%)
Purulent	2 (12%)

**Airway inflammation**	
Eosinophilia (>2.5%)	6 (35%)
Airway neutrophilia (>15%)	15 (88%)

**Clinical classification and management (*n* = 22)**	
Self-identified asthma (on follow-up Q)	10 (46%)

**Asthma diagnosed at clinical review**	
Intermittent asthma	4 (18%)
Persistent asthma	8 (36%)

**Optimization of medication and change of management plans**	13 (59%)

*^a^Mean (SD)*.

*^b^% predicted and IQR (25, 75)*.

*n = 1 child away for clinical examination. n = 1 child excluded as did not have medical records. Results are for only those children who completed clinical review missing (ever breathless, n = 1 (4%), ever wheeze, n = 1 (4%); ever hemoptysis, n = 1 (4%); ever tired/lethargic, n = 2 (9%), ever breathless 12 months, n = 2 (9%), ever wheeze 12 months, n = 2 (9%); ever hemoptysis 12-months, n = 2 (9%); ever chest pain, n = 2 (9%), ever tired/lethargic 12 months, n = 2 (9%), ever breathless 12 months, n = 2 (9%), ever wheeze 12 months, n = 2 (9%); ever hemoptysis 12 months, n = 2 (9%); ever chest pain, n = 2 (9%), ever tired/lethargic 12 months, n = 2 (9%), breathless now, n = 3 (13%), wheeze now, n = 3 (13%); hemoptysis now, n = 3 (13%); chest pain now, n = 3(13%), tired/lethargic now, n = 3 (13%), mum smoked(not stated, n = 10 (44%), exposed to household smoke, n = 7 (30%), previous respiratory hospitalization, n = 1 (4%), birth weight, n = 5 (23%), ICU admission, n = 1 (4%). mucoid (n = 13 (59%), sputum type; n = 13 (59%)*.

Over 20% of students reported current symptoms of breathlessness, wheeze, chest pain, and tiredness/lethargy (Table 4). Cough was reported in all students, with a dry cough 19 (86%) being most common.

Clinical diagnosis of asthma (intermittent or persistent) was higher 12 (54%) than student self-reported asthma 10 (46%). Eight of the eleven students with known asthma pre-evaluation had persistent asthma (Table 4). Optimization of asthma medications and change of management was required in 59% of students. Importantly, one student was suspected to have bronchiectasis and was referred for further investigation.

#### Tests Results

All students completed FE_NO_, spirometry, and AHR challenge. Induced sputum was successfully collected from 17 (77%) students. Clinically important high FE_NO_ levels (≥35 ppb) were documented in five (23%) students. Fifteen (88%) students had clinically important airway neutrophillia (>15% neutrophils) and 6 (35%) had airway eosinophilia (>2.5%). Of the five with FeNO levels ≥35 ppb, the median FeNO was 47 (max = 109), three were known to have asthma pre-evaluation, and airway eosinophilia was present in one. Of the 15 students with airway neutrophilia, five were known to have asthma pre-evaluation. Four (18%) students had abnormal spirometry results, but AHR was not present in any student.

A blood sample was collected from 14 (64%) students, and all results were within the normal ranges for cell counts. Absolute counts are as follows: Neutrophils: median 4 (IQR 3, 5); Eosinophils: median 0.4 (IQR 0.2, 0.7); and C-reactive protein: mean 1.3 (SD 0.7). The median peripheral eosinophil count in those with airway eosinophilia was 0.55 (IQR 0.3, 0.7).

## Discussion

Our study involving 203 students from two schools with a high percentage of Indigenous students showed that the ASPP was a feasible program for implementation into schools in the NT. For the clinical evaluation component, we found that students with respiratory symptoms had clinically important airway inflammation (35% airway eosinophilia and 88% neutrophilia) and clinically important elevated FE_NO_ levels (23% with values ≥35 ppb). Most students (59%) also required optimization of medication and management.

Our feasibility study established that the ASPP was well received in the urban NT schools. This was evidenced by (i) support from the NT Department of Education, (ii) successful implementation of the ASPP within school’s curriculum with minimal disruptions to existing curricula, (iii) positive feedback from Peer Leaders of the acceptability of the ASPP, (iv) teacher endorsement and willingness to be involved in future implementation of the program, and (v) obtaining a 70% response rate for follow-up questionnaires at 3 months. This study illustrates the potential for further school-based health interventions in at-risk groups.

Previous smoking was reported by 10% of students, with first time smoking when aged less than 12 years in 8% of students. These data are consistent with anecdotal evidence from several remote NT Indigenous communities, reporting that smoking is initiated among children aged less than 13 years. Students in our study reported high exposure to tobacco smoke at home (63%), far exceeding nationally reported data (7.8%) (2). This is consistent with our previous study on children hospitalized for asthma, which reported high exposure to tobacco smoke at home (Indigenous 95.2% and non-Indigenous 45.7%) (35). Our results highlight the importance of integrating interventions during adolescence to target social norms, behaviors, and attitudes (36) to prevent the uptake of tobacco smoking and prevent poorer long-term outcomes [e.g., chronic respiratory diseases such as bronchiectasis (35) and cardiovascular disease] (37).

Our sample was too small to examine for differences for improved asthma control and reduced uptake of tobacco smoking, and our study was not designed to replicate the results of previous studies (16, 17). However, we identified several important findings, including high self-reported asthma (19%) compared to the national average of 10.2% (2). Of those students with asthma, more than 20% reported being symptomatic in the previous 12 months, which is consistent with data reported from the Australian National Young People and Asthma Survey (38). Thus, our study that is focused on individuals, confirms the need to improve the diagnosis and management of respiratory symptoms in this age group.

Of students who underwent clinical evaluation, those with asthma had either poorly controlled asthma and/or were incorrectly diagnosed with asthma. Although no one had elevated systemic inflammatory markers, a majority had clinically important airway inflammation [88% had clinically important airway neutrophillia (>15%) and 35% had airway eosinophilia (>3%) (32, 33)]. Our findings of the lack of systemic makers in the presence of clinically important lower airway inflammation is in contrast to data in adults but consistent with pediatric studies (39, 40). We (41, 42) and others (39, 40) have shown the discordance between local (i.e., airways) and systemic inflammation in children with chronic lower airway infection and asthma, respectively. The possible reasons for this discordance include the lack of spill over effect in children (compared to adults) and the cross-sectional study design. However, these remain speculative and an in-depth discussion is beyond the scope of this paper. The reason for our findings of a very high proportion with airway neutrophilia is unknown, but possibly related to various factors, including tobacco smoke exposure, acute, and/or chronic persistent lower airway infection (1–3). Tobacco smoking in people with asthma is a known risk factor for poor asthma control and non-eosinophilic airway inflammation (21, 43). Eosinophilic inflammation identified in both FE_NO_ and sputum was very high, in particular, for those with previously diagnosed asthma, resulting in over two-thirds requiring optimization of medication and change of management. These data suggest that better individualized clinical evaluation is required and a larger community-based study is needed to ascertain objective diagnoses in a setting of high prevalence of chronic disease (e.g., bronchiectasis) to improve clinical outcomes particularly for Indigenous Australians. Our cohort study on children referred to respiratory specialists for chronic cough found that 70% were previously diagnosed incorrectly with asthma before referral (44). Also, Indigenous children were significantly more likely to have radiologically proven bronchiectasis (odds ratio = 4.4, 95% CI 1.9, 10) than non-Indigenous children on further evaluation. As respiratory issues account for the most common reason why Indigenous Australians present to doctors and the second most common self-reported chronic illness (45), it is imperative that better individualized management is required.

Our results are also consistent with national data describing 91% of Australian youth with asthma reporting poor control, with 63% reporting being short of breath on a weekly basis (38). In addition, 11.6% reported being current smokers (38). Plausible factors for suboptimal management in this group may include independent decision making of adolescents, reduced role of caregivers, less medical supervision, decreased adherence to therapy, and peer pressure (46). Further, individuals from low socioeconomic backgrounds are at particular risk for poorer outcomes, as found by a recent study evaluating risk factors for asthma-related deaths in children (47). Earlier studies have shown that the proportion of Indigenous children with poorly controlled asthma is higher than Australia-wide data, and that management was generally suboptimal (48).

Our study has several limitations. Firstly, the numbers of students were lower than anticipated. We had planned to include 200 Grade 7 students; however, with the withdrawal of a large school shortly before commencing the ASPP, we had limited time to find another school, which was much smaller. Secondly, one school required parental consent for objective measurements (e.g., eCO test and questionnaires). Thirdly, the smoking pledge was inconsistently done with Peer Leaders in the fourth lesson of the program. Nevertheless, valuable lessons were learnt that will support future implementation of the ASPP include (i) while we received high level school support, earlier involvement with class teachers is needed to encourage support of Peer Leaders during lessons, (ii) negotiating opt-out consent will provide more robust data to further strengthen findings from previous studies (16, 17), and (iii) questionnaires were not suitable for this population and further modification is required to make them more literacy and language appropriate. Using electronic devices such as tablets to complete questionnaires will restrict the possibility of conflicting responses, in addition to having a more reliable dataset to compare against objective measurements such as eCO levels. It could be argued that the withdrawal of the third school meant that the ASPP program may not feasible in the NT. However, we do not believe this is the case and would not have impacted the outcomes of this feasibility pilot study for several reasons. First, we were planning to undertake the study in two schools only, however, when one school was unable participate in the study due to logistical reasons at the school level, which were not related to the study, we had to recruit another smaller school in the vicinity, which also had high proportions of Indigenous students. Second, we have shown the feasibility of the ASPP in two diverse schools in Darwin as discussed above. Importantly, the implementation of the program model has shown to be effective in other geographically diverse populations in rural and metropolitan regions in Australia and in Jordan (15, 17, 24).

In conclusion, implementation of the ASPP in two urban-based NT schools was feasible and well received by the NT Department of Education and Children’s Services and schools. Of the students reporting current respiratory symptoms, most were found to have clinically significant airway inflammation and suboptimal management. Therefore, better community-based data, improving asthma management and preventing uptake of tobacco smoking of adolescents through innovative programs such as the ASPP in schools could potentially improve lung health particularly of Indigenous Australians.

## Author Contributions

GM setup and managed the study, recruited participants, cleaned the data, performed the data analysis, and wrote the manuscript. AC and SS conceptualized the study. AC, SS, and JS obtained the grant and edited the manuscript. CW recruited participants, schools, contributed to data collection, and edited the manuscript. HP and SP contributed to data collection, processing sputum samples, and edited the manuscript. AC and SCS undertook the clinical evaluation.

## Conflict of Interest Statement

The authors declare that they have no conflicts of interest relevant to this article to disclose.

## Abbreviations

ASPP, Asthma and Smoking Prevention Project; ACT, Asthma Control Test; eCO, exhaled carbon monoxide test; CO, carbon monoxide in breath; IQR, interquartile range; NT, Northern Territory; QoL, quality of life; Triple A, adolescent asthma action.
